# Effect of resistant compartment on pathogen strategy in partially migratory populations

**DOI:** 10.1371/journal.pone.0316640

**Published:** 2025-02-25

**Authors:** Cynthia Shao, Martha Torstenson, Allison K Shaw

**Affiliations:** 1 Department of Biochemistry, University of Minnesota Twin Cities, Minneapolis, Minnesota, United States of America; 2 Department of Ecology, Evolution and Behavior, University of Minnesota Twin Cities, St. Paul, Minnesota, United States of America; Penn State Health Milton S Hershey Medical Center, UNITEDSTATES OF AMERICA

## Abstract

Migration, the recurring movement of animals between habitats, can exert pressures on the pathogens they host. Properties of host populations can determine pathogen strategy (e.g. virulence) to increase pathogen fitness. To study the effect of adding a resistant compartment on virulence evolution, we developed an SIRS model and examined the winning pathogen strategy across different rates of recovery and of immunity loss. We find that when hosts spend a relatively long time in the resistant compartment, a more virulent pathogen evolves. These results have implications in conservation of migratory animal populations afflicted by disease.

## Introduction

Understanding the factors that shape host-pathogen interactions is critical for both science broadly as well as for specific applications to animal, plant and human health challenges [1–3]. Two key aspects of host-pathogen interactions are virulence and transmission rate. Although there are many definitions of virulence in the literature [4,5], we define virulence as disease-induced host mortality [6].

Furthermore, there is often a tradeoff between transmission and virulence: high transmission rates come at the cost of increased host mortality, reducing the lifespan of the host and decreasing opportunity to spread, while pathogens that reproduce more slowly can do so at lower cost to the host, which increases the timespan in which a pathogen can spread but results in a lower transmission rate [7]. More specifically, the relationship between virulence and transmission is often convex where the curve of transmission as a function of mortality is concave down [8,9].

One factor that can shape pathogen transmission and virulence is host movement. Both theoretical and empirical work suggests that virulence should increase when infection can occur across long distances (i.e., as host movement increases) while parasites should be less virulent when transmission is local [10,11]. However, theory also predicts that virulence should increase with natural host mortality, since this tends to decrease the duration of infection [12]. Since increased movement often comes with the cost of increased mortality [13], increased movement by hosts could also reduce infection duration, thus leading to decreased virulence. A similar tension has been found in studying pathogen virulence in spreading host populations. When the only effect of parasite infection was to decrease host survival, the most virulent parasites were favored [14,15]. However, if parasite infection simultaneously reduced host movement, lower virulence evolved [15]. However, the bulk of our knowledge of host movement comes from host dispersal, which is only one form of movement. In contrast, relatively less is known about how other forms of host movements like seasonal migration (recurring movement of animals between habitats) shape transmission and virulence.

Although much of the work exploring host-pathogen interactions in migratory species has focused on how pathogens shape host migration [16], we are increasingly learning about how host seasonal migration can shape pathogen virulence [17]. Recent theory shows that sedendary and migratory hosts can favor different pathogen virulence strategies [18]. Furthermore, properties of host populations can determine which pathogen strategies lead to the highest pathogen fitness [19]. For example, both the degree of tolerance that hosts have to pathogens as well as host pace of life can shape pathogen strategy [18].

However, this theory is based on only one of the possible types of host-pathogen systems [20,21], with other yet to be explored. Specifically, the above model was an SIS (susceptible - infected - susceptible) compartmental model, based on a host-pathogen system where hosts that recover from infection can be immediately reinfected (i.e., no long-lasting immunity). In contrast, many pathogens are better described by an SIR (susceptible – infected - resistant) model where recovered hosts gain immunity in the resistant compartment [22]. Migration itself can actually facilitate recovery from infection (migratory recovery; [23,24]) so migrants and residents may differ in their recovery rates, which could be relevant for pathogen trait evolution. Finally, resistant individuals may not have long lasting immunity and thus, might move back into the susceptible compartment to possibly be infected again, a dynamic that is captured by SIRS (susceptible - infected - resistant - susceptible) model. Since recovered and resistant individuals cannot immediately be re-infected, we expect that including a resistant compartment can affect pathogen evolution and virulence. Whether or not immunity is acquired is an especially important consideration for migratory species, since migrants may be favored to reduce immune function during migration [25].

Existing theory also typically starts with a host species that is fully migratory or fully non-migratory (i.e., resident). In contrast, for many migratory species across taxa (mammals, fish, birds, invertebrates), only some individuals migrate while others do not; a phenomenon termed partial migration [26]. There are a number of factors that favor partial migration over full migration or full residency. These include state or condition-dependent differences (e.g., age, dominance) across individuals that lead to different costs and benefits to migrating, avoiding high density (which can bring increased competition, predation, and disease), and if individuals benefit by accumulating resources for several years before migrating to reproduce [27,28].

Here, we develop a model to understand how the presence of a resistant compartment affects pathogen evolution in a partially migratory host population. Specifically, we developed an SIRS model that included pathogen strategies of different virulence levels and transmission rates. We quantified how the best pathogen strategy varies with the rate of immunity loss and other key model parameters. This represents a ‘Tinkerer’ approach to developing theory [29] where we explore whether our understanding of a theoretical outcome changes when we add a new element (i.e, immunity) to a model.

## 1 Methods and model

### 1.1 Overview

The model [30] investigates infection dynamics and pathogen strategy with an SIRS model. The host population consists of both migrants and residents, and we study how differences between the two groups (e.g. cost of migration, differing recovery rates during part of the year) affect infection dynamics. We investigate pathogen strategy by creating three strains of the pathogen with different virulence levels. We simulated the number of individuals in each compartment of the SIRS model for 2000 years then, at the end of each simulation, we determined which pathogen strategy was most common (i.e., most successful). We ran simulations across different values of the infection cost of migration, rate of immunity loss, and rate of recovery in habitat 1, in order to better understand the relationship between pathogen strategy and host migration strategy.

### 1.2 Compartments

**Fig 1:**
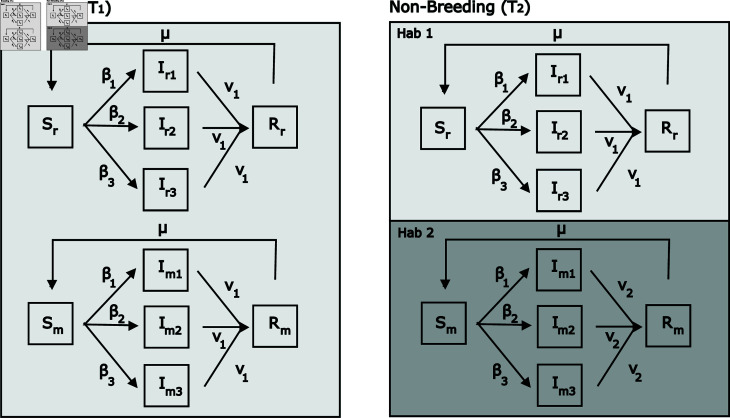
Model schematic. Migrants move between Habitat #1 and Habitat #2 while residents stay in Habitat #1 year-round. Recovery rate is $\nu_{1}$ in Habitat #1 and $\nu_{2}$ in Habitat #2. See Table 1 for parameter values.

We created an SIRS model with three infection classes (Fig 1). Infection class 1 was the least virulent, infection class 2 was intermediately virulent, and infection class 3 was the most virulent. These three infection classes provide a sufficient range of virulence levels to understand qualitatively how increasing virulence affects the behavior of the model. The host population was split into migrants and residents. There are 10 total classes in the model: susceptible residents ($S_{r}$), class 1 infected residents ($I_{r_{1}}$), class 2 infected residents ($I_{r_{2}}$), class 3 infected residents ($I_{r_{3}}$), resistant residents ($R_{r}$), susceptible migrants ($S_{m}$), class 1 infected migrants ($I_{m_{1}}$), class 2 infected residents ($I_{m_{2}}$), class 3 infected residents ($I_{m_{2}}$), and resistant migrants ($R_{m}$). Each class started with an initial population of 100 individuals. Migrants can move between the migrant compartments but cannot become residents and vice versa. However, migrants and residents affect each other through both pathogen transmission and density dependence (which is based on the total population of migrants and residents).

### 1.3 Transmission rate and virulence trade-off

Pathogens often experience a trade-off between transmission rate and virulence, as the cost of increasing replication within the host (associated with more transmission) is increased host mortality. Transmission rate and virulence have a convex relationship [8]. Thus, as a pathogen’s virulence increases, the marginal increase in transmission associated with a given increase in virulence lowers. Pathogens with low transmission rates thus have lower virulence than pathogens with high transmission rates. Thus, pathogens with low transmission rates keep their hosts alive for longer and have a longer amount of time to transmit to new hosts. A highly virulent pathogen would infect individuals at a higher rate than a less virulent pathogen but would kill its host more quickly and thus have less time to proliferate and infect another host. In our model we describe transmission rate as


$$\begin{array}{ccc} \beta_{i}=c_{A}\sqrt{c_{B}+\alpha_{i}} & & \end{array}$$ (1)

where $\alpha_{i}$ is the disease-induced mortality rate of pathogen strain *i* (the added mortality from being infected), and $c_{A}$ and $c_{B}$ are constants. We set $c_{A}$ to 0.005 to ensure a reasonable transmission rate where susceptible individuals were infected at a rate that would not overwhelm the population with infected individuals, and set $c_{B}$ to 0.14 which captures the amount of baseline transmission that a pathogen can experience before imposing a mortality cost on their host. Each infected class had a unique transmission rate dependent on the virulence level of the class. Transmission rate was the same for migrants and residents infected by the same infection class of pathogen.

### 1.4 Loss of immunity

We set up the model so that it could be run as either an SIR model (setting parameter *μ* = 0 defined below) or as an SIRS model (setting parameter 0 < *μ* < 1). For *μ* = 0, the model is effectively an SIR model with no movement from the resistant compartment to the susceptible compartment except for births. For *μ* > 0, some percentage of the resistant compartment loses resistance and moves back into the susceptible compartment in an SIRS model. As *μ* increases, individuals move out of the resistant compartment more quickly, and in the extreme the model effectively becomes an SIS model.

### 1.5 Winning pathogen strategy

In conflict situations, an evolutionarily stable strategy (ESS) is a strategy adopted by the majority of the population such that another competing strategy is unable to confer higher reproductive fitness [31]. We take a similar approach in our model, by competing pathogen strategies with different virulence levels to determine the ‘winning’ strategy within the host population. We explored under which conditions each pathogen strategy wins. When an initial host population includes equal proportions of individuals infected with each pathogen class, the ‘winning’ pathogen strategy corresponds to the infection compartment that makes up the largest proportion of the final population. This often means that the other two pathogen strategies have population sizes that are  ~ 0.

### 1.6 Partial migration

We set up the host population with migrant and resident individuals (i.e, with the potential for partial migration). During part of the year ($T_{1}$), migrants and residents are in the same habitat and can infect each other. This is also the period of the year where births occurred. During the remainder of the year ($T_{2}$), residents stay in the same habitat while migrants move to a separate habitat and return at the end of $T_{2}$. We assume $T_{1}\;+\;T_{2}=1$, i.e., migration is instantaneous in that we do not explicitly model infection dynamics during transit. When they are in separate habitats, residents can only be infected by residents and migrants can only be infected by migrants. Transmission rates and disease-induced mortality rates were the same between residents and migrants infected with the same class of pathogen. The recovery rate (movement from an infection compartment to a resistant compartment) was higher for migrants ($\nu_{2}$ in Habitat #2) than residents ($\nu_{1}$ in Habitat #1) during $T_{2}$, in order to simulate migratory recovery. At the end of each simulation, we determined whether there were more migrants or residents.

### 1.7 Equations

We describe our model with several sets of equations, detailed below. The meaning and values of all parameters are given in Table 1.

**Table 1:** Model symbols, meanings, and default values.

Symbol	Meaning	Default value
$T_{1}$	Time spent in one habitat	0.5
$T_{2}$	Time spent in two habitats	0.5
*b*	Birth rate	2
*d*	Pathogen-independent mortality	0.14
$\alpha_{1}$	Class 1 disease-induced mortality	0.01
$\alpha_{2}$	Class 2 disease-induced mortality	0.05
$\alpha_{3}$	Class 3 disease-induced mortality	0.1
$\beta_{i}$	Class *i* Transmission rate	from Eqn 1
*σ*	Disease-induced fecundity cost	–0.02
*γ*	Density dependence parameter	0.0001
$\delta_{S}$	Susceptible cost of migration	0.001
$\delta_{I}$	Infected cost of migration	varied
$\delta_{R}$	Resistant cost of migration	0.001
*μ*	Immunity loss rate	varied
$\nu_{1}$	Recovery rate in habitat 1	varied
$\nu_{2}$	Recovery rate in habitat 2	$\nu_{1}$ + 0.12
$c_{A}$	Scaling transmission rate constant	0.005
$c_{B}$	Baseline transmission rate constant	0.14

#### One habitat ( $T_{1}$ ).

During time $T_{1}$ (when migrants and residents are both in the same habitat), the dynamics of susceptible residents is given by


$$\begin{array}{llll} \frac{dS_{r}}{dt} & =\overset{3}{\underset{i=1}{\sum }}-S_{r}\beta_{i}(I_{r_{i}}+I_{m_{i}})+\mu R_{r}\; & & \;\\ & \;+\left[b(S_{r}+R_{r})+(b+\sigma )\overset{3}{\underset{i=1}{\sum }}I_{r_{i}}\right](1-\gamma N)-dS_{r} \end{array}$$ (2)

and the dynamics of susceptible migrants by


$$\begin{array}{llll} \frac{dS_{m}}{dt} & =\overset{3}{\underset{i=1}{\sum }}-S_{m}\beta_{i}(I_{r_{i}}+I_{m_{i}})+\mu R_{m}\; & & \;\\ & \;+\left[b(S_{m}+R_{m})+(b+\sigma )\overset{3}{\underset{i=1}{\sum }}I_{m_{i}}\right](1-\gamma N)-dS_{m}\;. \end{array}$$ (3)

The first term in each equation describes how individuals move out of the susceptible compartment (to the *I* compartments) by becoming infected according to transmission rate $\beta_{i}$, given by Eqn (1). Susceptible individuals can be infected by any infected individual (regardless of whether they are a migrant or resident). The second term describes how individuals move in to the susceptible compartment (from the *R* compartments) when immunity is lost, at rate *μ*. The third term describes reproduction where *b* is the density-independent birth rate, *σ* is the disease-induced fecundity cost, *γ* is the strength of density-dependence in reproduction, and *N* is the total host population size (summed across both migrants and residents). The last term in each equation describes pathogen-independent mortality at rate *d* (the rate at which individuals die naturally). We set *d* to 0.14, so that there was some mortality but the population was able to grow and persist in the absence of the pathogen. We kept the birth rate (*b*) constant at 2, which was high enough to ensure population growth (compared to *d*) but not high enough to overwhelm the population with susceptible individuals. We set *γ* to 0.0001 to have a carrying capacity of 10,000 (to be relatively higher than our starting population of 1,000 individuals). As the total population *N* increases towards the carrying capacity, the term (1 - *γN*) decreases towards 0 and decreases the number of individuals born. Infected individuals produced less offspring due to the effects of the disease. All newborn individuals are susceptible.

The dynamics of infected residents and migrants are given by


$$\begin{array}{ll} \frac{dI_{r_{i}}}{dt} & =\beta_{i}S_{r}(I_{r_{i}}+I_{m_{i}})-\nu_{1}I_{r_{i}}-(d+\alpha_{i})I_{r_{i}} \end{array}$$ (4)

$$\begin{array}{ll} \frac{dI_{m_{i}}}{dt} & =\beta_{i}S_{m}(I_{r_{i}}+I_{m_{i}})-\nu_{1}I_{m_{i}}-(d+\alpha_{i})I_{m_{i}}\;. \end{array}$$ (5)

The first terms in each equation describe how susceptible individuals become newly infected. We assume that already infected individuals cannot become infected with other pathogen strains (i.e. no coinfection). The second terms describes infected individuals that recover at rate $\nu_{1}$. The third term describes mortality, both from natural causes and from disease, at rate $d\;+\;\alpha_{i}$. We chose values for the disease-induced mortality rates ($\alpha_{1},\alpha_{2},\alpha_{3}$) that captured a wide range of possible detrimental effects of the pathogen while also allowing the population to reach steady state in a long enough time frame to investigate the model’s behavior.

The dynamics of resistant residents and migrants are given by


$$\begin{array}{ll} \frac{dR_{r}}{dt} & =\nu_{1}(I_{r_{1}}+I_{r_{2}}+I_{r_{3}})-\mu R_{r}-dR_{r} \end{array}$$ (6)

$$\begin{array}{ll} \frac{dR_{m}}{dt} & =\nu_{1}(I_{m_{1}}+I_{m_{2}}+I_{m_{3}})-\mu R_{m}-dR_{m}\;. \end{array}$$ (7)

Here, the first terms describe individuals that have recovered from infection. The second term describes resistant individuals that lose immunity and move back to the susceptible compartments. The final term describes pathogen-independent mortality, as above.

#### Two habitats ( $T_{2}$ ).

During time $T_{2}$ (when migrants and residents are in two separate habitats), the dynamics of susceptible residents and migrants is given by


$$\begin{array}{ll} \frac{dS_{r}}{dt} & =\overset{3}{\underset{i=1}{\sum }}-\beta_{i}S_{r}I_{r_{i}}+\mu R_{r}-dS_{r} \end{array}$$ (8)

$$\begin{array}{ll} \frac{dS_{m}}{dt} & =\overset{3}{\underset{i=1}{\sum }}-\beta_{i}S_{m}I_{m_{i}}+\mu R_{m}-dS_{m}\;. \end{array}$$ (9)

The dynamics are similar to those during $T_{1}$ (Eqns 2 and 3) except that residents are only infected by other residents, and migrants are only infected by other migrants, and no reproduction occurs. The dynamics of infected residents and migrants is given by


$$\begin{array}{ll} \frac{dI_{r_{i}}}{dt} & =\beta_{i}S_{r}I_{r_{i}}-\nu_{1}I_{r_{i}}-(d+\alpha_{i})I_{r_{i}} \end{array}$$ (10)

$$\begin{array}{ll} \frac{dI_{m_{i}}}{dt} & =\beta_{i}S_{m}I_{m_{i}}-\nu_{2}I_{m_{i}}-(d+\alpha_{i})I_{m_{i}}\;. \end{array}$$ (11)

We assume that recovery rate is habitat-dependent, so infected residents recover at rate $\nu_{1}$ while infected migrants recovers at a higher rate $\nu_{2}$. We varied $\nu_{1}$ and set $\nu_{2}=\nu_{1}\;+\;0.12$ to ensure a noticeable benefit for migrants who survive the process of migration. Finally, the dynamics of resistant residents and migrants are given by


$$\begin{array}{ll} \frac{dR_{r}}{dt} & =\nu_{1}(I_{r_{1}}+I_{r_{2}}+I_{r_{3}})-\mu R_{r}-dR_{r} \end{array}$$ (12)

$$\begin{array}{ll} \frac{dR_{m}}{dt} & =\nu_{2}(I_{m_{1}}+I_{m_{2}}+I_{m_{3}})-\mu R_{m}-dR_{m}\;, \end{array}$$ (13)

which is identical to the dynamics during $T_{1}$ (Eqns 6 and 7).

We assume that migrants experience a mortality cost each time they migrate. To capture this cost, twice a year (at the end of $T_{1}$ and $T_{2}$), we multiply the number of susceptible migrants by $1\;-\;\delta_{S}$, the number of infected migrants by $1\;-\;\delta_{I}$, and the number of resistant migrants by $1\;-\;\delta_{R}$, so the actual number of individuals is


$$\begin{array}{ll} S_{m} & =(1-\delta_{S})S_{m} \end{array}$$ (14)

$$\begin{array}{ll} I_{m_{i}} & =(1-\delta_{I})I_{m_{i}} \end{array}$$ (15)

$$\begin{array}{ll} R_{m} & =(1-\delta_{R})R_{m}. \end{array}$$ (16)

The susceptible and resistant cost of migration ($\delta_{S}$ and $\delta_{R}$) were set to 0.001 to ensure there was some detrimental consequence to migrating, but not enough to consistently favor the residents for all parameter combinations we considered.

### 1.8 Simulations

We started each simulation with 100 individuals in each of the 10 classes ($S_{r},S_{m},I_{r_{1}},I_{m_{1}},I_{r_{2}}$, $I_{m_{2}},I_{r_{3}}$, $I_{m_{3}},R_{r},R_{m}$) and ran it for 2000 years. For each annual cycle we first simulate the one habitat Eqns (2–7) for $T_{1}$ time, then apply mortality via Eqns (14–16) to the 5 classes that migrate, then simulate the two habitat Eqns (8–13) for $T_{2}$ time, then finally apply mortality via Eqns (14–16) again to the 5 classes that migrate. Then we repeat these steps for each annual cycle. We ran simulations under different values of the immunity loss rate (*μ*), the recovery rate in habitat 1 ($\nu_{1}$) and the infected cost of migration $\delta_{I}$ in order to explore interactions between migration dynamics and pathogen strategy. We kept the remaining parameters constant. For each simulation, we quantified the number of individuals by pathogen strategy and by host migration strategy.

## 2 Results

**Fig 2:**
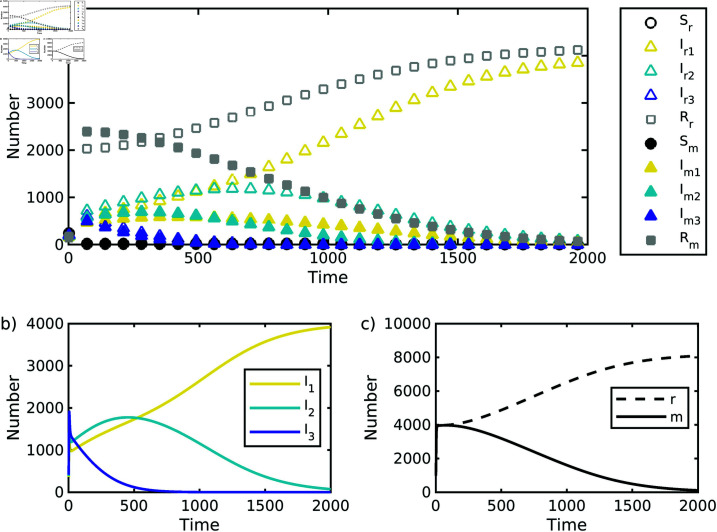
Example of a single simulation output. The number of host individuals of each type over time for (a) all 10 types: *S* suceptible, *I* infected, and *R* recovered individuals: for *r* residents and *m* migrants, (b) the number of individuals by pathogen class (1, 2, or 3), (c) the number of individuals by host strategy (residents or migrants). Parameter values: $\delta_{S}=\delta_{R}=0.001$, $\delta_{I}=4\delta_{S}$, $\nu_{1}=0.14$, $\nu_{2}=\nu_{1}+0.12$; and *μ* = 0. Other parameter values given in Table 1.

We can examine the output of each simulation run of our model in a number of ways. We tracked the total number of individuals of each of the 10 host types over time – the number of susceptible, infected (with each of the three pathogen strains) and recovered individuals for each migrant and resident hosts (Fig 2a). We determined which pathogen strategy won by combining the number of migrant and resident hosts infected with each pathogen strain; typically we found that a single pathogen strategy was much more abundant than the other two (Fig 2b). We also determined what happened to the host types by combining the number of hosts within each the migrant and host categories; again we typically found that a single host strategy (migrant or resident) was more abundant than the other (Fig 2c).

**Fig 3:**
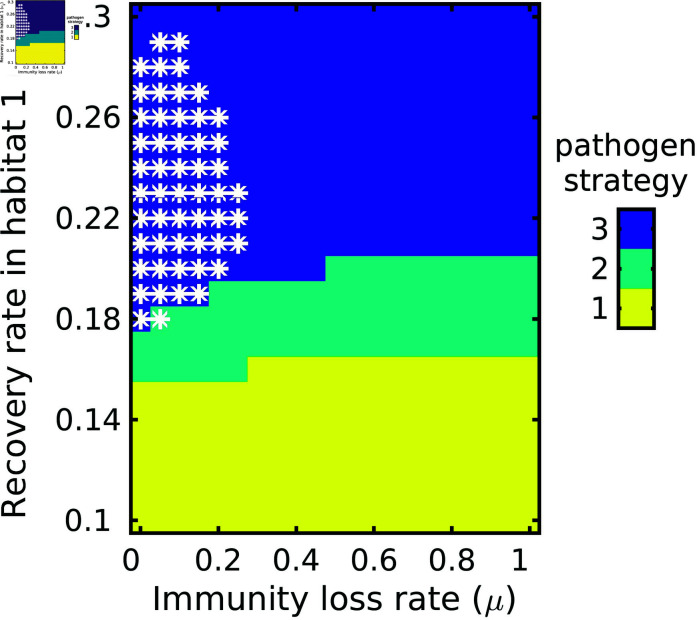
Winning pathogen strategy. The pathogen class (1, 2 or 3) that wins as a function of the immunity loss rate (*μ*; x-axis) and recovery rate ($\nu_{1}$; y-axis). Parameter values: $\delta_{S}=\delta_{R}=0.001$, $\delta_{I}=4\delta_{S}$, $\nu_{2}=\nu_{1}+0.12$; *μ* and $\nu_{1}$ were varied. Other parameter values given in Table 1. Asterisks indicate where there were more migrants than residents.

**Fig 4:**
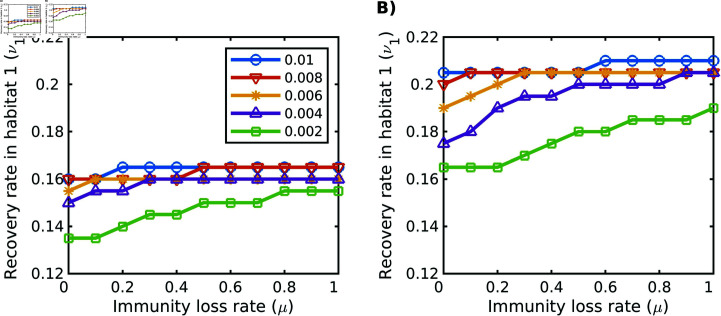
Transitions between winning pathogen strategies. The recovery rate ($\nu_{1}$; y-axis) at which the winning infection class changes **A)** from class 1 to class 2, and **B)** from class 2 to class 3, as a function of the immunity loss rate (*μ*; x-axis), for different values of $\delta_{I}$ (colors), the infection cost of migration. Parameter values: $\delta_{S}=\delta_{R}=0.001$, $\nu_{2}=\nu_{1}+0.12$; *μ*, $\nu_{1}$, and $\delta_{I}$ were varied. Other parameter values given in Table 1.

Our results show that there are cases where each of the three pathogen strategies can win, i.e. infect the most hosts in the population (Fig 3). Which pathogen strain did best in our model depended in large part on how quickly hosts recovered from infection. In particular, pathogen strategy 1 (which has the lowest virulence) was favored when recovery rate ($\nu_{1}$) was relatively low, while pathogen strategy 3 (which has the highest virulence) was favored when $\nu_{1}$ was high (Fig 3). Thus, increasing recovery favored an increase in pathogen virulence. To a lesser extent, the pathogen strategy that did best also depended on how quickly host immunity was lost, where faster rates of immunity loss (*μ*) favored less virulent pathogens (Fig 3). These results generally hold across all values of $\delta_{I}$ (the cost of migration for infected individuals) that we considered (Fig 4).

### 2.1 Resistant compartment mediates interaction between recovery and immunity loss rates

To understand how the existence of a resistance class affects virulence, we can compare our results across different parameter values. For $\nu_{1}=0$ (no recovery) the model is effectively an SI model: individuals can get infected but never recover. This favors low pathogen virulence. For $\nu_{1}>0$ but *μ* = 0, the model is effectively an SIR model: individuals recover but never lose immunity. Here, the virulence of the pathogen strategy increases as $\nu_{1}$ increases. Finally, for $\nu_{1}>0$ and *μ* > 0, we have an SIRS model. By varying the relative values of $\nu_{1}$ and *μ*, we vary the average amount of time that hosts spend immune once they recover from infection. A high *μ* value and low $\nu_{1}$ value mean that hosts recovery slowly and lose immunity quickly, thus spending little time in the R compartment. In contrast a low *μ* value and high $\nu_{1}$ value mean that hosts recovery quickly and lose immunity slowly, thus spending a longer time in the *R* compartment. Looking diagonally across Fig 3, we can see that spending longer in the *R* compartment (moving from the lower right to the upper left) favors increased pathogen virulence.

### 2.2 Interaction between migration and virulence

In our model, infected individuals face a trade-off when it comes to migration: infected individuals have a higher cost of migration than susceptible and resistant individuals, but gain the benefit of increased recovery while in the migratory habitat (since $\nu_{2}>\nu_{1}$). Which pathogen strain did best in our model thus also depended in part on how costly migration was for infected individuals. We can plot the values of $\nu_{1}$ and *μ* where we see a switch from pathogen strategy 1 to pathogen strategy 2 being favored (Fig 4). Doing this, we see that when the cost of migration for infected individuals ($\delta_{I}$) is high, the switch from strategy 1 to strategy 2 occurs for higher values of $\nu_{1}$ and *μ* than if $\delta_{I}$ is low (Fig 4). In other words, as the infection-based cost of migration increases, less virulent pathogens are favored. Finally, we also typically found that there were more residents than migrants, except when *μ* was quite low and $\nu_{1}$ was high. The switch between these two regions indicates the point at which the benefit of increased recovery rate in the migratory habitat overcomes the cost of migration.

## Discussion

### Effect of host migration

Here, we developed a model to understand how the presence of a resistant compartment, combined with host migratory behavior, affected pathogen evolution. Our first finding was that host migration can indeed shape pathogen virulence. Specifically we found that a lower cost of migration (e.g. a lower $\delta_{I}$ in our model) increases pathogen virulence (Fig 4), presumably by increasing the proportion of migrants within the population.

This result parallels theory on other forms of host movement (i.e. dispersal). Past work finds that increased host dispersal favors pathogens that enter and exit the host quickly [32]. Similarly, past theory has found that populations with restricted spatial movement evolve lower virulence, and populations with greater connectivity evolve higher virulence [10,33]. This result is also found empirically in bacteriophage systems, where less virulent strains are favored when movement is restricted [33].

The relative number of migratory and resident hosts that emerged also varied in our model across the parameter values we considered. When the cost of migration for infected individuals is sufficiently low and the recovery rate for migrants is sufficiently high, migrants are able to win over the residents. This is likely because migrants are only able to maintain their population under circumstances with low cost of migration and high recovery. We found that conditions where the migrant population was greater than the resident population, were also conditions where a more virulent pathogen strain won (Fig 3). In contrast to this finding, some empirical studies on butterfly populations found that parasites from resident winter-breeding sources had the same virulence as parasites from migratory sources, while other studies found that increased transmission opportunities featured increased virulence [34]. Theoretically, an increased proportion of migrants could lead to reduced virulence due to migratory escape and migratory culling [34]. In a sense, we see this result in our model as well. Although we do not have migratory escape, we do have migratory culling (the degree to which $\delta_{I}$ is bigger than $\delta_{S}$ or $\delta_{R}$). As migratory culling increases ($\delta_{I}$ gets bigger), we see a shift towards less virulent pathogens (Fig 4), which may be driven by hosts shifting to becoming non-migratory.

### Effect of resistant compartment

Our second finding was that the presence of a resistant compartment–and how long hosts spent in that compartment–shaped pathogen virulence. In an SIR model (*μ* = 0), a pathogen with a high recovery rate may favor a high virulence strategy, as the pathogen will have less time to reproduce in the host and will aggressively use the host’s resources. A low recovery rate may favor a low virulence strategy, as the pathogen can stay in the host longer to proliferate and infect other possible hosts. However, in SIRS models where resistant individuals can lose immunity and move back into the susceptible compartment, the strength of this trend may be reduced, thought the direction of the effect remains consistent. If there is a steady supply of susceptible individuals to infect, the pathogen may favor a high virulence strategy regardless of recovery rate, as killing the host rapidly is less of a concern if there are many susceptible individuals to infect. We see this reflected in Figs 3 and 4, where higher recovery rates favor higher virulence strains.

The rate of immunity loss, *μ*, moves individuals from the resistant compartment to the susceptible compartment. A higher *μ* means that a population has fewer resistant individuals and more susceptible individuals compared to a lower *μ*, where the population would consist of more resistant individuals and fewer susceptible individuals. A population with a smaller proportion of susceptible individuals could favor a less virulent strain, as slowly expending host resources to spread the pathogen would be preferable to killing the host quickly when there are not many individuals to infect at a given point in time. Waiting in the host means the pathogen can infect more individuals. By increasing the number of susceptible individuals when having resistant individuals lose immunity, the size of the susceptible compartment increases. However, pathogen strategy itself also impacts the number of susceptible individuals present. Highly virulent pathogens reduce the number of resistant hosts (i.e. by killing off infected hosts before they recover), which otherwise would block transmission [35]. When recovery and immunity loss are both high, the most virulent pathogen strain wins (Fig 3), as the pathogen has a continuous supply of susceptible hosts to infect and the infected individuals do not stay infected for long, meaning the pathogen will rapidly exploit the host’s resources.

### Parallels to past work

We can draw a number of parallels between our findings here and past work about the evolution of pathogen virulence. First, consider the classic metric in disease ecology *R*_0_, the basic reproduction ratio, i.e., the product of how fast an infected host generates new infections and the average duration of infection. Classically it was thought that pathogens should always evolve to maximize *R*_0_ [6]. However, more recent work has demonstrated that this is often not the case across a range of different ecological scenarios, including spatial structure of hosts [36]. In our model, infected migrants and residents experience both different recovery rates during the part of the year they are apart as well as different mortality rates (due to increased mortality from migration). Thus, infection duration differs across hosts, which in turn leads to a different pathogen strain having the highest *R*_0_ in residents versus migrants. Overall then, the spatial structure in our model, combined with environmental heterogeneity (i.e., different recovery rates in different environments) mean that the pathogen strategy that is favored is not necessarily the one that maximizes *R*_0_. A related point is that typically *R*_0_ is independent of the rate of immunity loss and thus changing loss of immunity should not affect the favored pathogen strategy. Yet in our model, changing immunity loss rate shifts the relative costs and benefits of migration, leading to a different relative composition of migrants and residents in the population, and thus a different best pathogen strategy (Fig 3).

We can also compare our results to recent work on evolution of pathogen virulence within migratory host populations. Shorter infection durations should typically lead to more virulent pathogen strategies. In this paper, we found that shorter infection durations can be caused by higher recovery rates. In past work, we have shown that shorter duration caused by higher mortality also leads to a more virulent pathogen strategy [18]. Relatedly, hosts that are better adapted to cope with pathogen infection can lead to higher virulence. This can occur e.g. via hosts maintaining immunity for longer (lower immunity loss rate *μ* in our model), or can occur if hosts tolerate infection more (i.e. experience lower mortality) [18]. Finally, the abundance of susceptible individuals available to become infected has a stronger effect on virulence than loss of infected individuals. Here we see this result in that loss of immunity by recovered individuals matters more than recovery of infected individuals (Fig 4). In past work we found that fecundity matters more than mortality in shaping pathogen virulence [18].

### Future directions

Future work could explore different sources of density dependence. A model with mortality-based density dependence could provide further insight into population dynamics. For fecundity-based density dependence (as implemented in this model), the birth rate decreases when the population nears carrying capacity and mortality rate is held steady. For mortality-based density dependence, the mortality rate increases when the population nears carrying capacity and birth rate is held steady. For fecundity-based density dependence (like our model), if resistant individuals make up the majority of the population and the population nears the carrying capacity, then the number of susceptible individuals decreases (due to a decreasing birth rate). On the other hand, for mortality-based density dependence mortality rate would increase among all compartments and the rate at which new susceptible individuals enter the population through fecundity will not be affected by host population density. Thus, models that employ a mortality-based density dependence approach may generate different results than the ones we found here with a fecundity-based density dependence approach.

## Conclusion

Our results have implications for conservation in partially migratory animal populations. Building understanding of how pathogen resistance and loss of resistance can affect virulence evolution in a partially migratory species can aid in animal populations experiencing a disease outbreak.
